# Validity and Reliability of a Short Form of the Questionnaire for the Reflective Practice of Nursing Involving Invasive Mechanical Ventilation: A Cross-Sectional Study

**DOI:** 10.3390/nursrep13030101

**Published:** 2023-09-01

**Authors:** Makoto Tsukuda, Atsuko Fukuda, Junko Shogaki, Ikuko Miyawaki

**Affiliations:** 1 College of Nursing Art and Science, University of Hyogo, 13-71 Kitaoji-Cho, Akashi 673-0021, Hyogo, Japan; 2 Graduate School of Health Sciences, Kobe University, 7-10-2 Tomogaoka, Suma-Ku, Kobe 654-0142, Hyogo, Japan

**Keywords:** invasive mechanical ventilation, validity, reliability, questionnaire, short form

## Abstract

The number of patients on ventilators is rapidly increasing owing to the coronavirus pandemic. The previously developed Questionnaire for the Reflective Practice of Nursing Involving Invasive Mechanical Ventilation (Q-RPN-IMV) for the care of patients on ventilators includes nurses’ thought processes as items. This study aims to develop a short form of the Q-RPN-IMV for immediate use in practice and to test its reliability and validity. A convenience sample of 629 participants was used to explore the factor structure using factor analysis. The test–retest reliability was assessed using the intraclass correlation coefficient (ICC). The study was a cross-sectional design instrument development study and was reported according to GRRAS guidelines. Q-RPN-IMV short form was divided into ventilator management and patient management. The ventilator management comprised 31 items organized into six factors. Cronbach’s alpha ranged from 0.82 to 0.91, and the ICC ranged from 0.82 to 0.89. The patient management comprised 27 items organized into five factors. Cronbach’s alpha ranged from 0.75 to 0.97, and ICC ranged from 0.75 to 0.97. The Q-RPN-IMV short form is a reliable and validated instrument for assessing care for patients on ventilators. This study was not registered.

## 1. Introduction

The essential skills required for nursing practice for patients on ventilator support include knowledge of ventilators, invasive techniques for preventing complications, and risk prediction for safe management [1,2]. Therefore, management in the intensive care area is generally recommended for patients on ventilator support because it is well-equipped and has qualified experts to manage such patients [3]. Recently, the number of patients with respiratory diseases due to aging has increased worldwide. Moreover, the number of patients with severe respiratory symptoms due to the spread of coronavirus disease 2019 (COVID-19) is rapidly increasing [4,5,6]; thus, the number of patients on ventilators during treatment has inevitably increased. In the current scenario of the ongoing pandemic, intensive care units cannot exclusively manage patients on ventilators. Therefore, the general ward continues to serve as an urgent management site for such patients [7,8,9,10].

Respiratory therapists in Europe and the United States are coping with the pressures of a growing number of patients suffering from infectious diseases; however, in the event of a pandemic, there could be a worldwide shortage of ventilators and specially trained respiratory therapists [11,12]. General nurses from various backgrounds would therefore be forced to manage patients on ventilators and provide in-hospital and home care, including oxygen therapy, respiratory assistance, and ventilator therapy [13]. Furthermore, even if nurses have little experience caring for patients on ventilators, they are expected to have the minimum knowledge and skills necessary to safely manage these patients. Therefore, nurses in charge of patients requiring ventilators carry a heavy burden because of a substantial amount of responsibility and potential lack of experience, frequently leading to negative emotions, such as anxiety, stress, and lack of self-confidence [9].

Despite this situation, inexperienced nurses currently provide ventilator education to other nursing staff using the methods of the facility. Furthermore, most nursing education programs generally solely focus on imparting knowledge on the individual skills of ventilator care [14]. During the pandemic, it was difficult to ensure the quality and safety of nursing care for patients on ventilators because conducting group training in the hospital was impossible due to the increased number of patients. Therefore, with the rapid increase in the number of patients on ventilators, there is a need for an evaluation sheet that allows inexperienced nurses to provide safe nursing care to patients on ventilators and for nurses who practice on such patients to look back on their practice and self-evaluate it.

In a previous study, we developed the Questionnaire for the Reflective Practice of Nursing Involving Invasive Mechanical Ventilation (Q-RPN-IMV), a comprehensive, itemized measure of nursing practice of ventilator care by professional and certified nurses who are considered experts in fields related to ventilator care, including their thought processes [15]. This questionnaire differs from previous checklists because it is not simply a checklist to determine whether self-care has been practiced. However, because ventilator care is complex and includes advanced-level practice items practiced by specialized and certified nurses, the number of items is notably large, and the so-called “Q-RPN-IMV long form” could not be used routinely by inexperienced nurses. Even for inexperienced nurses to safely manage ventilator-implanted patients and to be able to use the questionnaire immediately in clinical practice, the number of items in the questionnaire must be carefully selected and consolidated. Therefore, we carefully selected suitable items from the Q-RPN-IMV developed in previous studies and created a short form that can be easily used by inexperienced nurses. This study aims to develop a short form of the Q-RPN-IMV, based on the Q-RPN-IMV, for immediate use in practice and to test its reliability and validity.

## 2. Materials and Methods

### 2.1. Study Design

Scale development was based on the guidelines described in Scale Development: Theory and Applications and the Consensus-based Standards for the Selection of Health Status Measurement [16]. Moreover, we followed the Guidelines for Reporting Reliability and Agreement Studies (GRRAS) [17].

### 2.2. Setting and Participants

Using convenience sampling, we selected clinical nurses who had experience caring for patients on ventilators. To meet the criteria for good methodology, a sample size of at least seven times the number of survey items and an absolute number of at least 100 are required [18]. The first version of the questionnaire contained 71 items; therefore, the minimum sample size was 497. The purpose of this study was explained in writing and orally to the facility director to be surveyed, and permission was obtained before the start of the study. A questionnaire on ventilator care was distributed to clinical nurses working in wards where ventilators were used. Criteria for participation were full-time employment in a general hospital with at least 500 beds that was considered to offer group training on ventilators. Certified nurses, nursing managers, licensed practical nurses/nursing assistants, and midwives were excluded from the study. Nurses’ responses to the questionnaire were collected from April 2020 to December 2021. The nurses were asked to post their responses in a collection box set up in the ward within 1 week after distribution. Furthermore, the participants were asked to answer a questionnaire on ventilator care after 10–14 days for test–retest reliability to examine the stability of the study. A filled-in questionnaire implied the nurses’ consent to participate in the study [19].

### 2.3. Instrument

Participants answered a questionnaire consisting of the following three sections: (1) demographic characteristics, (2) the Self-Evaluation Scale for Nursing Involving Invasive Mechanical Ventilation, and (3) the educational needs assessment tool for clinical nurses.

#### 2.3.1. Participant Demographic Characteristics

From the participants, we collected data on age, clinical career (years of work experience), number of patients on ventilators received to date, hospital affiliation, and presence or absence of certified nurse support.

#### 2.3.2. Self-Evaluation Scale for Nursing Involving Invasive Mechanical Ventilation

To investigate the participants’ nursing practice with patients on ventilators, we utilized the nursing practice process items related to ventilator care required by clinical nurses from the Q-RPN-IMV. The Q-RPN-IMV is a questionnaire that was itemized by observing the respiratory care for patients on ventilators practiced by certified nurses in previous studies, including their thought processes. Furthermore, the Q-RPN-IMV comprises 136 items: 26 observation, 66 judgment, and 44 implementation items. These items were relevant to the respiratory care of patients on ventilators by certified nurses. From these 136 items, the most essential were selected for immediate use by inexperienced nurses.

Content validity refers to whether the content of the scale adequately captures the constructs and requires expert judgment. The content validity index (CVI) is the most widely reported content validity approach in instrument development and can be calculated using Item-CVI (I-CVI) and Scale-Level-CVI (S-CVI). To verify content validity, the Delphi method was performed three times by 20 certified nurses, and the items were selected by more than 80% of the experts on all three occasions [20]. Furthermore, those items were classified by an expert panel of certified nurses into two groups to make them easier to understand by less experienced nurses; items were classified into two groups, “ventilator management” and “patient management”. After reviewing the items, they were classified into “ventilator management” and “patient management”, and 71 items were adopted as proposal items. Thus, a ventilator evaluation scale consisting of 32 items for machine management and 39 for patient management was proposed for 71 items. Each item was rated on a 5-point Likert scale from 1 (not at all) to 5 (always) to determine the frequency of implementation. The resulting questionnaire was pretested on five nurses in a general ward, and the superficial validity of the proposed scale was examined by checking for unclear expressions and the time required for easy comprehension, even by inexperienced nurses.

### 2.4. Data Analysis

In the statistical analysis, questionnaires with missing items were excluded, and item analysis was performed first. Subsequently, exploratory factor analysis (EFA), confirmatory factor analysis (CFA), and criterion-related validity were performed to assess the scale’s validity. Finally, internal consistency and reproducibility were examined to assess the reliability. All statistical analyses were performed using IBM SPSS version 28 and Amos 28 (IBM Corp., Armonk, NY, USA).

#### 2.4.1. Item Analysis

Standard deviations and means for all items were estimated to detect ceiling and floor effects.

#### 2.4.2. Validity Analysis

EFA was conducted to determine the factor structure supporting “ventilator management” and “patient management” of patients on ventilators. To assess the suitability of the data for factor analysis, the Kaiser–Meyer–Olkin (KMO) test for sampling adequacy and Bartlett’s specificity test were performed [18]. The number of factors was visually inspected using scree plots to identify the ideal number of potential factors and to determine if the KMO criterion of an eigenvalue ≥ 1 was met, and the proportion of variance explained by each factor [21]. Maximum likelihood methods with promax rotation were then performed [22]. Items with factor loadings < 0.4 were excluded [23]. As a rule, the sum of the variance explained should be greater than 50% [24]. EFA was conducted again with the removed items (Items with factor loadings < 0.4). The number of factors was determined to include all items with factor loadings of 0.40 or greater. For items with approximate factor loadings, a valid factor analysis was conducted using a covariance structure analysis, considering the item content included in that factor.

The CFA was performed to test the model fit. For the CFA, we used maximum-likelihood estimation and evaluated model fit. Model fit was tested based on the χ^2^, normed χ^2^ (χ^2^/df), comparative fit index (CFI), and root mean square error of approximation (RMSEA) index used to assess the fit of the scale. A χ^2^/df value of ≤3 is considered adequate. Furthermore, the fit was considered good if the CFI value was >0.90 and the RMSEA value was <0.08 [25,26].

#### 2.4.3. Reliability Analysis

Reliability was verified using Cronbach’s alpha coefficients for the entire scale and each factor. To examine stability and verify the test–retest reliability, we determined the intraclass correlation coefficient (ICC) of the response scores to the scale obtained at the two-time points [19]. ICCs were determined as <0.50 (poor), 0.50–0.80 (fair to good), and >0.80 (excellent) [27].

### 2.5. Ethical Consideration

Participants were informed of the purpose and methods of the study and the risks and benefits of participation. In addition, participants were informed that their participation was voluntary, that they were free to drop out at any time, and that their privacy would not be affected. Data were collected anonymously using assigned IDs and were strictly controlled. Data handling and disposal procedures were also explained. In the interest of transparency, it was further explained that some of the findings would be presented in a public forum. Researchers provided participants with contact information. Ethical approval was obtained from the Institutional Review Board (IRB) Ethics Committee of the author’s university (No. 696). All methods were performed in accordance with relevant guidelines and regulations.

## 3. Results

### 3.1. Participant Characteristics

Table 1 presents the participant characteristics. Questionnaires were distributed to 945 clinical nurses working in general hospitals with more than 500 beds and with experience working with patients on ventilators, and 754 returned the questionnaires.

Of the 754 returned questionnaires, 125 with >20% missing data were excluded, and 629 questionnaires were included in the analysis. The questionnaire collection rate was 79.8%, and the effective response rate was 83.4%. For the stability study, 157 questionnaires were collected, and 122 were analyzed after excluding 35 with missing data. The number of nurses with less than 5 years of experience was 212 (33.7%), which is higher than the percentage of nurses in Japan, but this is because the survey excluded managers. Approximately half of the participants were responsible for more than six ventilators.

### 3.2. Item Analysis

Each of the 71 items was analyzed. The mean individual item scores ranged from 3.03 to 4.89 (standard deviation 0.44–1.53), with no ceiling or bottom effects.

### 3.3. Factor Analysis

EFA was performed on managing ventilator items (32 items) and patient items (39 items) using the main factor method and promax rotation.

The following results were obtained according to the conditions for factor selection.

#### 3.3.1. Ventilator Management

The KMO measure of sampling adequacy was 0.90. There were six factors based on eigenvalues ≥ 1 and the scree criterion. Items with a loading value of ≥0.4 were retained, and item 12 was deleted because the factor loadings were <0.4. The six factors covered the following roles: initial confirmation, artificial airways, alarm management, humidification management, emergency management, and airway fixation. The contributions of factors 1–6 were 32.29%, 10.79%, 6.03%, 5.36%, 4.91%, and 3.75%, respectively, and the cumulative contribution was 63.13%. The model fit index for the scale was calculated using CFA, and the results showed that χ^2^ = 2058.1, χ^2^/df = 4.91, CFI = 0.844, and RMSEA = 0.079 (Table 2). The model fit was not optimal, although it was acceptable with respect to the factor contribution ratio.

#### 3.3.2. Patient Management

The KMO measure of sampling adequacy was 0.90. There were five factors based on eigenvalues ≥1 and the scree criterion. Items with a loading value of ≥ 0.4 were retained, and item 12 was deleted because the factor loadings were <0.4. These five factors covered the following roles: prevention of complications, prevention of VAP, safe transfer, skin management, and post-transfer assessment. The contributions of factors 1–5 were 31.81%, 12.87%, 6.13%, 5.44%, and 4.18%, respectively, and the cumulative contribution was 60.43%. The model fit index for the scale was calculated using CFA, and the results showed that χ^2^ = 1723.9, *p* < 0.01, χ^2^/df = 5.49, CFI = 0.841, and RMSEA = 0.085 (Table 3). Factor contributions were acceptable. Model coefficients were not good, despite attempts to improve the model index as much as possible by removing items.

### 3.4. Reliability Testing

The Cronbach’s alpha coefficient for “Ventilator Management” was 0.91 at the scale level and 0.82, 0.82, 0.89, 0.87, 0.87, and 0.82 for factors 1–6, respectively (Table 2). In contrast, for “Patient Management”, the coefficients were 0.91 at the scale level and 0.88, 0.86, 0.75, 0.97, and 0.77 for factors 1–5, respectively (Table 3). For reproducibility, the ICC intraclass correlation coefficient for factors, calculated using data from the 152 participants who returned their test–retest responses, ranged from 0.75 to 0.97 (Table 4). All factors of ventilator management show high reliability. Patient management factors 1, 2, and 4 showed high reliability. Factors 3 and 5 showed acceptable reliability. A lower ICC was predicted because the frequency of practice increased as nurses reflected on ventilator care in their nursing practice through retesting.

## 4. Discussion

The basis for this study was the Q-IMV-RPN, developed from the existing literature and observations of actual skilled nursing practice, which include their thought processes concerning the nursing practices required for ventilator care. The rating scale was developed by selecting essential items that should be used immediately by nurses in general wards. However, the nursing skills to be provided to patients with respiratory distress are complex. Therefore, for each machine and patient management, essential nursing practices were consolidated according to the nursing process, and a scale was created.

Mechanical management of ventilator care in a general hospital ward comprises six factors. These included essential information, such as setting alarms and damaging the ventilator circuit, which should be checked at regular intervals and personnel shifts. Factor 1, “initial confirmation”, included confirming the forced ventilation mode setting, volume of single breaths, and respiratory frequency. Factor 2, “artificial airway”, included an assessment to prevent incidents related to artificial airways that occur most frequently in general wards [28] and an assessment related to humidification. Factor 3, “alarm management”, included an assessment of the patient’s situation when the alarm went off. This is a crucial factor because even if alarms are set, it is impossible to prevent or manage abnormalities in the patient without being able to assess what is happening during the alarm [29]. Factor 4 was related to the confirmation and implementation of “humidification management”. Heating and humidification are essential assessment items for ventilator-associated pneumonia (VAP) prevention; however, they are also important factors, as inadequate management can lead to serious incidents, such as airway burns. Factors 5 and 6 included the confirmation and implementation items related to the artificial airway [30]. Therefore, it is obvious that machine management for ventilator care includes the management of machine settings and alarms and the nursing practice process related to the management of the artificial airway, including heating and humidification.

Management of patients in a general hospital ward essentially comprises five factors. Factor 1, “complication prevention”, consists of items for implementing necessary care, such as intraperiod suctioning, repositioning, and oral care, to prevent pneumonia and unscheduled extubation. We believe that the essential care items that should be implemented for patients using ventilators are concentrated in this category. Factor 2, “prevent VAP”, comprises a collection of items describing the perspectives and rationale for preventing VAP, such as repositioning, oral care, and endotracheal suctioning. Therefore, it should be practiced continuously in conjunction with factor 1. Factor 2, the perspective of assessment, and factor 1, the implementation of safe care, can be regarded as practices to prevent complications [31]. Factor 3, “transfer safely”, includes items such as checking the connection and setting the ventilator. Many incidents related to ventilators in general wards involve unscheduled extubation during transfer, poor aftercare connection, and incorrect ventilator settings. Therefore, it is considered a crucial factor in safety management [28]. Factor 4, “skin management”, concentrates on items related to skin damage caused by the fixation of the artificial airway. Generally, great emphasis should be placed on preventing the unscheduled removal of artificial airways. Therefore, there is a high likelihood of skin damage due to immobilization. Tracheal tube fixation is essential for safe breathing. However, skin damage from fixation can prevent stable fixation from being maintained, increase patient distress, and reduce the patient’s quality of life after extubation. Therefore, we value this factor as a perspective for implementing care while maintaining safety. Factor 5, “assessment post-transfer”, consolidates items related to the assessment and the rationale for factor 3. Factors 3 and 5 together can be considered to pertain to safety management practice. Patient management for ventilator care focuses on nursing practices to prevent VAP and the unscheduled removal of the artificial airway. This is also expected to occur during the transfer of the patient from one location to another before and after care. The content pertains to nursing care aiming to prevent skin damage and improve safety management.

In Japan, nurses control the use of ventilators. However, this is not covered in basic nursing education. Instead, nurses are trained in the field of practice while working in hospitals. However, during the pandemic, the number of patients on ventilators increased. Moreover, there was no time to train nurses who could care for patients on ventilators because they were faced with urgent life-and-death situations. Therefore, we believe that the scale developed in this study is highly beneficial for inexperienced nurses to manage ventilators safely. Furthermore, nurses’ ability to practice ventilator care can be assessed using this scale, which can further help evaluate the ability of wards to manage ventilators. Moreover, managers can evaluate which wards can safely manage patients on ventilators removed from the intensive care unit during trying times. It can also be used for objective evaluation by a third party to assess ventilator management capabilities and to help in education. This scale itemizes the nursing practice process of ventilator care that should be practiced by nurses, including thought processes. Therefore, it can benefit nurses with limited experience to safely practice ventilator care. Simultaneously, it can be used to assess the practice and educational impact of group education on ventilator care and which items should be taught effectively. Therefore, it is possible to evaluate the developmental stages of nurses using this scale. Furthermore, this scale can help hospital administrators evaluate which nurses can practice ventilator management and to what extent it can be used as a material to consider which area to move patients with ventilators. As there has been a rising need for human resources to provide ventilator care, it is believed that safe care for patients on ventilation can be provided using this system with the currently used checklist.

Regarding the item content, the items were narrowed down to the minimum necessary while verbalizing the practice of professional nurses, including their thought processes. Furthermore, the scale was divided into ventilator management and patient management so that routine nurses and inexperienced nurses could safely practice ventilator care. Therefore, this scale can be used to self-evaluate one’s nursing practice and as an evaluation tool for others for educational purposes. In particular, we believe that, in cases where actual practical exercises or group training could not be conducted, including pandemics, the scale created in this study will allow even novices to practice the minimum necessary ventilator care and to reflect on the content of their practice.

However, this study had several limitations. First, the scale was developed only for Japanese nurses. Therefore, whether this scale can be used in other countries needs to be examined. Second, this study included only generalists working in Japanese hospitals with 500 or more beds. Therefore, future studies investigating its applicability to nurses working in smaller hospitals are needed. Third, if pediatric patients had participated in this study, it is highly likely that their attitudes and methods toward ventilator management would have changed. Therefore, the model as a scale is not optimal and needs to be improved. In the future, the number of items for each target nurse should be considered, and the structure should be balanced. In addition, it is necessary to create a scale that can be adapted according to the target patients.

## 5. Conclusions

The short form developed in this study is based on the Q-RPN-IMV, and the items are carefully selected to provide the minimum necessary content for nursing practice professionals to care for patients on ventilators. Furthermore, to facilitate use by generalist nurses, the scale is divided into thirty-one items with six factors related to ventilator management and twenty-seven items with five factors related to patient management to ensure reliability and validity.

## Figures and Tables

**Table 1:** Participant characteristics (*n* = 629).

		*n*	%
Sex			
	Men	49	(7.8)
	Women	580	(92.2)
Nursing experience			
	<3 years	60	(9.5)
	<3−5 years	152	(24.2)
	<6−10 years	145	(23.1)
	>10 years	272	(43.2)
Number of patients requiring IMV			
	1	16	(2.5)
	2−3	63	(10.0)
	4−5	237	(37.7)
	6−10	61	(9.7)
	>10	252	(40.1)
Support from professional			
	Yes	516	(82.0)
	No	113	(18.0)

Data are presented as *n* (%). IMV: invasive mechanical ventilation.

**Table 2:** Ventilator Management (Cronbach’s α = 0.91).

Domain/Item Number and Content				Factor Loadings					
				Factor 1	Factor 2	Factor 3	Factor 4	Factor 5	Factor 6
Factor 1		Initial confirmation (Cronbach’s α = 0.82)							
	16	At the start of a shift, confirm the alarm settings for the artificial respirator together with another staff member.		0.76	−0.08	−0.18	−0.06	−0.01	−0.09
	18	Confirm that the power is plugged into an outlet that is either red or brown.		0.71	−0.08	0.01	0.03	0.00	−0.03
	19	Confirm the patient’s respiratory data displayed on the artificial respirator (number of breaths, volume of air per breath, volume of air per minute, maximum respiratory tract internal pressure) every two hours.		0.69	−0.13	0.12	0.02	−0.07	−0.05
	21	In case the artificial respirator alarm rings, confirm the alarm message displayed on the LCD screen.		0.68	0.22	−0.03	−0.02	0.03	0.02
	17	When confirming the presence or absence of a circuit leak, confirm that each individual artificial airway connection and circuit component is connected properly by both sight and touch.		0.63	0.01	0.19	−0.06	−0.01	0.09
	15	At the start of a shift, confirm the artificial respirator instructions and settings together with another staff member.		0.59	−0.05	0.10	0.04	−0.11	0.15
	13	Confirm that a cannula of the same type and size as the one currently inserted in the tracheostomy tube, lubricant, and an 11 mL syringe are prepared on the patient’s bedside table		0.50	0.09	−0.04	0.03	0.08	−0.16
	11	Confirm cuff pressure at the start of each shift		0.48	−0.10	0.12	−0.01	0.06	0.07
	64	Ensure no contamination or damage to the artificial respirator circuit		0.45	0.38	−0.08	0.08	−0.02	0.02
Factor 2		Artificial airway (Cronbach’s α = 0.82)							
	32	In a situation where a patient’s sputum viscosity is low and a humidifier is in use, consider that to be a sign that the patient’s artificial nose needs to be replaced.		−0.20	0.89	−0.03	−0.03	−0.03	0.02
	33	In a situation where a patient’s sputum viscosity is high and an artificial nose is in use, consider that to be a sign that the patient needs to be switched to a humidifier.		−0.04	0.86	−0.10	0.03	−0.12	0.08
	26	Be aware that artificial noses and heated humidifiers must not be used at the same time.		0.25	0.56	−0.08	−0.03	0.11	−0.01
	14	If an accidental removal of the tracheotomy tube occurs, one should call a doctor, ventilate with VBM from the mouth, and prepare a tracheostomy tube of the same size and an emergency cart (prepare for intubation).		0.05	0.55	0.06	−0.07	0.22	−0.12
	6	In the event of accidental removal of the intubation tube, one should ventilate with VBM, prepare an emergency cart (prepare for intubation), and call a doctor.		−0.12	0.55	0.18	0.12	−0.04	−0.01
	31	Confirm the volume and characteristics of sputum by observing surface viscosity and consistency.		0.18	0.49	0.00	−0.02	−0.05	−0.06
	4	If oral care causes the fixation of the tracheal tube to loosen, reaffix it.		−0.21	0.46	0.27	−0.02	0.00	0.01
Factor 3		Alarm management (Cronbach’s α = 0.89)							
	23	If a “high pressure” alarm rings, confirm that there are no abnormal values for SpO_2_, pulse, blood pressure, single breath volume, or breath volume per minute and check that there is no accumulated sputum or blockage in the circuit.		−0.01	−0.02	0.96	−0.01	−0.06	−0.06
	25	If the “low pressure” alarm rings, since this indicates a possibility of a leak in the circuit, check the circuit and cuff pressure		0.04	0.01	0.81	0.02	0.00	−0.08
	24	If the “apnea” alarm rings, confirm the number of breaths and consult with a doctor about changing the ventilation mode setting and alarm setting as and when necessary.		0.03	0.00	0.76	−0.02	0.01	−0.02
	22	If a “high pressure” alarm rings, confirm the presence or absence of fighting or bucking, respiratory tract blockage due to secretions, or bending of the circuit.		−0.08	0.17	0.70	0.00	0.04	0.01
	20	Consider the alarms related to the artificial respirator as having a possibility of being directly linked to the lives of patients.		0.12	0.12	0.51	0.00	0.01	0.11
	10	Use cuff pressure to assess whether there is a possibility of damage and ulceration of the respiratory tract mucosa.		0.19	−0.06	0.45	−0.01	0.06	0.08
Factor 4		Humidification management (Cronbach’s α = 0.87)							
	28	Confirm that heated humidifiers are on.		−0.09	0.02	−0.05	0.98	0.07	−0.08
	27	Check and refill the water chambers of heated humidifiers to ensure the water does not run out.		−0.03	−0.11	0.02	0.90	−0.03	0.04
	30	Confirm that artificial noses are clean and free of contaminants and change them in case they are not.		0.11	0.13	0.00	0.61	−0.07	0.02
	29	Confirm that the water inside the water chambers of heated humidifiers is clean and free of contaminants.		0.17	0.01	0.04	0.59	0.06	0.04
Factor 5		Emergency management (Cronbach’s α = 0.87)							
	7	Confirm that the tracheostomy tube is attached at the midline.		−0.12	0.00	0.00	0.01	0.95	−0.01
	8	Attach the tracheostomy tube using the pressure of one finger on each side.		0.15	−0.09	−0.01	0.01	0.84	−0.05
	54	Confirm that the attachment band of the tracheal tube is not loose before and after adjusting a patient’s body position.		−0.03	0.05	0.02	0.01	0.54	0.25
Factor 6		Airway fixation (Cronbach’s α = 0.82)							
	2	Confirm that the tape holding the tracheal tube in place is not likely to unstick due to wetness or peeling.		0.01	−0.04	−0.07	−0.02	0.02	0.99
	1	Confirm that the tracheal tube is fixed in place at the corners of the mouth or confirm the measurement (in cm) from the incisors specified in the instructions.		−0.09	0.02	0.01	0.01	0.02	0.78
Contribution ratio				32.29	10.79	6.03	5.36	4.91	3.75
Cumulative contribution ratio					43.08	49.11	54.47	59.38	63.13
Factor correlations			Factor 1	-	0.20	0.54	0.49	0.46	0.47
			Factor 2		-	0.54	0.46	0.39	0.36
			Factor 3			-	0.47	0.48	0.55
			Factor 4				-	0.38	0.43
			Factor 5					-	0.46
			Factor 6						-
χ^2^ = 2058.1, *p* < 0.01, χ^2^/df = 4.91, CFI = 0.844, RMSEA = 0.079									
Factor extraction method: main factor method, motation method: Promax method with Kaiser normalization.									
Squared figures: factor loadings > 0.4.									
31 items from 32 initial items.									
The following items had low loadings and were excluded from the assessment:									
	12	Set cuff pressure to a range between 22 and 32 cmH_2_O.							

**Table 3:** Patient Management (Cronbach’s α = 0.91).

Domain/Item Number and Content				Factor Loadings				
				Factor 1	Factor 2	Factor 3	Factor 4	Factor 5
Factor 1		Complication prevention (Cronbach’s α = 0.88)						
	58	Make sure there is plenty of slack in the breathing circuit when adjusting a patient’s body position.		0.85	−0.02	−0.14	0.01	−0.03
	63	Except when contraindicated, make sure the head is always at an angle of at least 30 degrees.		0.82	−0.05	0.06	−0.06	−0.09
	36	Carry out oral care at least once during each shift.		0.73	−0.05	−0.09	0.03	0.02
	70	When moving patients to beds, stretchers, inspection tables, or operating tables, one staff member holds the tracheal tube in place with their hands.		0.69	0.01	−0.03	0.01	0.02
	39	Before oral care, apply suction to the upper cuff area, oral cavity, nasal cavity, and trachea in that order.		0.66	−0.07	0.13	−0.02	−0.04
	48	Use a suction tube insertion length that reaches about 1 to 2 cm from the tip of the tracheal tube, inserting until just before the bifurcation of the trachea.		0.65	0.02	−0.14	0.03	0.05
	41	For patients who have teeth, brush their teeth with a toothbrush.		0.61	0.13	−0.08	0.02	0.06
	40	As a rinsing liquid, use 50 mL or more of tap water administered in 3–5 mL units while applying suction.		0.46	0.13	0.15	−0.07	0.03
	5	If there is drooling, carry out intraoral suction at intervals of once every 30 min to 1 h.		0.43	0.00	0.30	0.10	−0.15
	59	Adjust a patient’s body position together with the help of another staff member.		0.41	0.13	0.30	−0.05	−0.03
Factor 2		Prevent ventilator-associated pneumonia (VAP) (Cronbach’s α = 0.86)						
	52	Consider that body position is affected by the movements of the diaphragm and the chest.		−0.11	0.92	−0.06	−0.02	0.00
	34	Consider that the self-cleaning function of an intubated patient’s oral cavity declines when the patient is intubated.		−0.14	0.69	0.21	0.11	−0.10
	44	Consider that accumulated secretions in the upper cuff area, oral cavity, and nasal cavity drop into the lungs as a result of coughing induced by tracheal suction.		0.10	0.68	−0.04	−0.08	−0.04
	62	Understand that there is a possibility of inducing VAP due to the backflow of stomach contents.		0.04	0.60	−0.01	0.02	0.12
	35	Understand that dental plaque contains a large amount of bacteria and cannot be removed without brushing.		0.04	0.60	0.09	−0.02	−0.11
	43	Consider it possible that pulmonary alveoli could collapse as a result of tracheal suction.		0.15	0.57	−0.09	−0.06	0.10
	51	Consider the possibility that changing body position can result in differences in the volume of air per breath.		0.01	0.56	−0.10	0.03	0.34
Factor 3		Transfer the patient safely from one location to the other (Cronbach’s α = 0.75).						
	69	Prior to transport, attach a test lung to the artificial respirator and conduct a startup inspection.		−0.02	−0.07	0.74	−0.04	0.12
	67	Confirm that portable artificial respirator circuits are properly connected.		0.03	−0.03	0.64	0.05	0.18
	68	When patients are being transported, prepare a back valve mask, oxygen tank, spare tracheotomy cannula set, and a transport-use monitor (which can monitor ECG and SpO_2_).		−0.08	−0.16	0.64	−0.05	0.15
	38	Adjust cuff pressure to between 22 and 32 cmH_2_O before and after oral care.		0.14	0.28	0.46	0.00	−0.10
	46	Record the volume and characteristics of sputum recovered from tracheal suction once per shift.		−0.07	0.28	0.41	0.06	−0.07
Factor 4		Skin management (Cronbach’s α = 0.97)						
	3	Confirm the presence or absence of redness and ulceration of the lips.		0.03	−0.06	0.06	0.98	0.00
	9	Confirm the presence or absence of maceration, redness, and ulceration in the skin surrounding the tracheostomy incision.		−0.03	0.01	−0.07	0.98	0.00
Factor 5		Assessment post transferred (Cronbach’s α = 0.77)						
	71	After changing the transport-use respirator and artificial respirator, check the settings, number of breaths, volume of air per breath, maximum intratracheal pressure, volume of breaths per minute, breathing sounds, number of breaths, SpO_2_, and subjective symptoms to confirm that they are as they were before the change.		0.13	−0.02	0.14	0.01	0.83
	66	Do not execute nursing care within 30 min before or after the transport of patients.		−0.23	0.03	0.20	−0.04	0.71
	65	Prior to moving a patient, assess whether they have stable breathing and circulation.		0.27	0.19	−0.08	0.05	0.46
Contribution ratio				31.81	12.87	6.13	5.44	4.18
Cumulative contribution ratio					44.68	50.81	56.25	60.43
Factor correlations			Factor 1	-	0.58	−0.01	0.40	0.36
			Factor 2		-	0.36	0.48	0.61
			Factor 3			-	0.25	0.29
			Factor 4				-	0.26
			Factor 5					-
χ^2^ = 1723.9, *p* < 0.01, χ^2^/df = 5.49, CFI = 0.841, RMSEA = 0.085								
Factor extraction method: main factor method, rotation method: Promax method with Kaiser normalization.								
Squared figures: factor loadings above 0.4.								
27 items from 39 initial items.								
The following items had low loading and were excluded from the assessment:								
	37	For orally intubated patients, carry out oral care together with another staff member.						
	42	Consider that tracheal secretions are affected by gravity.						
	45	For patients without restrictions, perform sputum drainage and adjust their position to an angle of at least 40 to 60 degrees alternating between the left and right sides to prevent atelectasis.						
	47	Record any changes in the volume or characteristics of sputum recovered from tracheal suction.						
	49	Complete suction within 15 s after inserting the suction catheter.						
	50	After tracheal suction, confirm breath sounds, number of breaths, SpO_2_, and subjective symptoms to confirm that all sputum has been removed.						
	53	Before adjusting a patient’s body position, remove water from the circuit.						
	55	Perform oral cavity suction prior to adjusting a patient’s body position.						
	56	When changing the orientation of the body or moving the neck, use hands to keep the tracheal tube base in place.						
	57	When adjusting a patient’s body position, position the breathing circuit lower than the tracheal tube to prevent water in the breathing circuit from flowing into the trachea.						
	60	Adjust the position of the pillows to prevent the patient’s neck from getting into an extended position.						
	61	Before and after touching artificial respirators or patients, sterilize hands with quick-drying alcohol (if there is visible dirt, wash hands with soap and water).						

**Table 4:** Intraclass correlation coefficient and 95% confidence intervals.

		ICC *	95%CI **	*p*-Value ***
Ventilator Management				
	Factor 1	0.816	0.794–0.837	<0.001
	Factor 2	0.823	0.800–0.843	<0.001
	Factor 3	0.886	0.871–0.899	<0.001
	Factor 4	0.869	0.852–0.885	<0.001
	Factor 5	0.868	0.849–0.885	<0.001
	Factor 6	0.823	0.793–0.849	<0.001
Patient Management				
	Factor 1	0.875	0.860–0.889	<0.001
	Factor 2	0.861	0.844–0.877	<0.001
	Factor 3	0.752	0.720–0.781	<0.001
	Factor 4	0.968	0.962–0.972	<0.001
	Factor 5	0.771	0.738–0.800	<0.001

ICC * = intra-class correlation coefficient; CI ** = confidence interval; *p*-value *** = the statistically significant reference.

## Data Availability

The datasets used during the current study are available from the corresponding author upon request.

## Public Involvement Statement

No public involvement in any aspect of this research.

## Guidelines and Standards Statement

This manuscript was drafted against the guidelines described in the *Scale Development: Theory and Applications* and the Consensus-based Standards for the Selection of Health Status Measurement. Moreover, we followed the Guidelines for Reporting Reliability and Agreement Studies (GRRAS).
